# Identification and Analysis of Glioblastoma Biomarkers Based on Single Cell Sequencing

**DOI:** 10.3389/fbioe.2020.00167

**Published:** 2020-03-05

**Authors:** Quan Cheng, Jing Li, Fan Fan, Hui Cao, Zi-Yu Dai, Ze-Yu Wang, Song-Shan Feng

**Affiliations:** ^1^Department of Neurosurgery, Xiangya Hospital, Central South University, Changsha, China; ^2^Department of Clinical Pharmacology, Xiangya Hospital, Central South University, Changsha, China; ^3^Department of Rehabilitation, The Second Xiangya Hospital, Central South University, Changsha, China; ^4^Department of Psychiatry, The Second People’s Hospital of Hunan University of Chinese Medicine, Changsha, China

**Keywords:** glioblastoma biomarkers, scRNA-seq, mRMR method, support vector machine, pericarcinomatous environment

## Abstract

Glioblastoma (GBM) is one of the most common and aggressive primary adult brain tumors. Tumor heterogeneity poses a great challenge to the treatment of GBM, which is determined by both heterogeneous GBM cells and a complex tumor microenvironment. Single-cell RNA sequencing (scRNA-seq) enables the transcriptomes of great deal of individual cells to be assayed in an unbiased manner and has been applied in head and neck cancer, breast cancer, blood disease, and so on. In this study, based on the scRNA-seq results of infiltrating neoplastic cells in GBM, computational methods were applied to screen core biomarkers that can distinguish the discrepancy between GBM tumor and pericarcinomatous environment. The gene expression profiles of GBM from 2343 tumor cells and 1246 periphery cells were analyzed by maximum relevance minimum redundancy (mRMR). Upon further analysis of the feature lists yielded by the mRMR method, 31 important genes were extracted that may be essential biomarkers for GBM tumor cells. Besides, an optimal classification model using a support vector machine (SVM) algorithm as the classifier was also built. Our results provided insights of GBM mechanisms and may be useful for GBM diagnosis and therapy.

## Introduction

Glioblastoma (GBM), with an annual incidence of 3.19 per 100,000, maintains the most common and aggressive primary adult brain tumor (Stupp et al., 2007, 2017; Chinot et al., 2014; Gilbert et al., 2014; Ostrom et al., 2016). Currently, the standard therapeutic regimen has been established, including surgical resection, followed by radiotherapy with concurrent chemotherapy (temozolomide), then followed by maintenance therapy (temozolomide for 6–12 months) (Stupp et al., 2005). However, the diffuse nature of GBMs makes it invariably recur after treatment, rendering local therapies invalid, because the migrating GBM cells outside of the neoplasm core are usually unaffected by local therapies and hence cause recurrence of GBMs (Darmanis et al., 2017). The mean disease-free survival is just over 6 months and the mean overall survival also remains gloomy, with an approximately 25% 2-year survival rate after diagnosis and a 5–10% 5-year survival rate (Stupp et al., 2005, 2017; Das and Marsden, 2013).

Tumor heterogeneity poses a great challenge to the treatment of GBM, which is determined by both heterogeneous GBM cells and a complex tumor microenvironment. It is critical important for researchers to understand how different types of GBM cells interact with neoplasm cells through profiling of different types of cell from cell population in paraneoplastic environment, as well as identifying the lineage and phenotypes (Darmanis et al., 2017). Verhaak et al. (2010) has proved bulk tumor sequencing methods were useful in generating classification schemas of GBM subtypes, but the heterogeneity of GBM was not unveiled in essence (Cancer Genome Atlas Research Network, 2008). Until recently, RNA profiling was limited to ensemble-based approaches, averaging over bulk cell populations. Therefore, the advent of single-cell RNA sequencing (scRNA-seq) enables the transcriptomes of great deal of individual cells to be assayed in an unbiased manner (Stegle et al., 2015) and has been applied in head and neck cancer (Puram et al., 2017), breast cancer (Bajikar et al., 2017), blood disease (Zhao et al., 2017), and so on. Patel et al. (2014) profiled 430 cells from five GBM patients using scRNA-seq and described inter-patient variation and molecular diversity of tumor cells within individual GBM patients. The diversities of GBM cells within tumors are responsible for cancer progression and finally result in treatment failure.

Currently, in order to improve future treatment options, an increasing number of researchers have focused on the targeted agents or genes (Liu et al., 2013; Xiao et al., 2014; Li et al., 2018). Furnari et al. (2007) have identified genetic molecular mechanisms in GBM patients: (1) dysregulation of growth factor signaling through amplification and mutational activation of receptor tyrosine kinase (RTK) genes; (2) activation of the phosphatidyl inositol 3-kinase (PI3K) pathway; and (3) deactivation of the p53 and retinoblastoma tumor suppressor pathways. Moreover, four distinct GBM subclasses, including neural, proneural (PGFRA/IDH1 events), classical (focal EGFR events), and mesenchymal (NF1 mutation and loss), were defined by gene expression studies from The Cancer Genome Atlas (TCGA) (Verhaak et al., 2010), which also found the majority of GBM neoplasms had abnormalities in the pathways (RB, TP53, and RTK) through projecting copy number and mutation data on these pathways, revealing that this is a crucial step for GBM pathogenesis. Apart from such researches focused on tumor or microenvironment, many studies analyzed the gene expression of immune cells in GBM via scRNA-seq. Muller et al. (2017) identified 66 new gene sets which can be applied as biomarkers (such as P2RY12, CD49D, and HLA-DRA) to distinguish the different lineages of the macrophage cell subsets.

In this study, based on the scRNA-seq results of infiltrating neoplastic cells in GBM, computational methods were applied to screen core biomarkers that can distinguish the discrepancy between GBM tumor and pericarcinomatous environment. The gene expression profiles of GBM from 2343 tumor cells and 1246 periphery cells were analyzed by maximum relevance minimum redundancy (mRMR) (Peng et al., 2005). Upon further analysis of the feature lists yielded by the mRMR method, 31 important genes were extracted that may be essential biomarkers for GBM tumor cells. Besides, an optimal classification model using a support vector machine (SVM) algorithm (Ding and Dubchak, 2001) as the classifier was also built.

## Materials and Methods

### The Single Cell Gene Expression Profiles of Tumor and Surrounding Tissues

We download the single cell gene expression profiles of 2343 cells of tumor core and 1246 cells of surrounding tissue from Gene Expression Omnibus (GEO) with accession number of GSE84465 (Darmanis et al., 2017). 23,460 genes were measured using Illumina NextSeq 500. Within each sample, we counted the number of expressed genes, i.e., the number of genes with mapped reads. The average number of expressed genes in each sample was 2,581. Our goal is to discriminate the 2343 tumor cells (positive samples) and 1246 surrounding cells (negative samples).

### The mRMR Ranking of Discriminative Genes

There have been many statistics methods for identifying the differentially expressed genes (DEGs). But these methods did not consider the relationships between genes. Usually, the number of DEGs was too large to apply as biomarker. Therefore, we adopted the information theory-based mRMR (minimal Redundancy Maximal Relevance) method (Peng et al., 2005) to overcome this problem. The mRMR method not only considers the associations between genes and samples, but also the redundancy between genes. If several genes are similar, only the most representative gene will be selected. This approach has been proven to be effective and has been widely used for many biomedical feature selection problems (Niu et al., 2013; Zhao et al., 2013; Zhou et al., 2015; Zhang et al., 2016; Liu et al., 2017), especially in single cell RNA-Seq analysis (Zhang et al., 2019). The sample size of single cell data was large and the gene expression was spare. It was easy to get too many redundant significant genes using traditional statistical based method, such as *t*-test. Therefore, the mRMR was suitable for analyzing single cell data to get small number of non-redundant biomarkers.

Let’s describe the method mathematically. All genes, selected genes, to be selected genes can be represented as Ω, Ω_*s*_, and Ω_*t*_, respectively. The relevance of gene g from Ω*_*t*_* with tissue type *t* can be measured with mutual information (*I*) (Sun et al., 2012; Huang and Cai, 2013):

(1) D=I⁢(g,t).

And the redundancy *R* of the gene *g* with the selected genes in Ω*_*s*_* are

(2) $$R=\frac{1}{m}\,\left(\sum_{g_{i}\in \Omega_{s}}I\,\left(g,g_{i}\right)\right)$$

The goal of this algorithm is to get the gene *g*_*j*_ from Ω*_*t*_* that has maximum relevance with tissue type *t* and minimum redundancy with the selected genes in Ω*_*s*_*, i.e., maximize the mRMR function

(3) $$\max_{g_{j}\in \Omega_{t}}\left[I\left(g_{j},t\right)-\frac{1}{m}\left(\sum_{g_{i}\in \Omega_{s}}I\left(g_{j},g_{i}\right)\right)\right]\;\left(j=1,2,\ldots ,n\right)$$

The evaluation procedure will be continued for *N* rounds, and all the genes will be ranked as a list

(4) $$S=\left\{g_{1}^{\prime },g_{2}^{\prime },\ldots ,g_{h}^{\prime },\ldots ,g_{N}^{\prime },\right\}$$

The index *h* reflects the trade-off between relevance with tissue type and redundancy with selected genes. The smaller index *h* is, the better discriminating power the gene has.

### The Single Cell GBM Biomarker Optimization

Based on the top 100 mRMR genes, we constructed 100 SVM classifiers and applied an incremental feature selection (IFS) method (Jiang et al., 2013; Li et al., 2014; Shu et al., 2014; Zhang et al., 2014, 2015) to identify the optimal number of genes as biomarker. The svm function from R package e10171 was used to implement the SVM method. Each candidate gene set $S_{k}=\left\{g_{1}^{\prime },g_{2}^{\prime },\ldots ,g_{k}^{\prime }\right\}\left(1\leq k\leq 100\right)$ included the top *k* genes in the mRMR list.

We used leave-one-out cross validation (LOOCV) (Cui et al., 2013; Yang et al., 2014) to evaluate the prediction performance of each SVM classifier. During LOOCV, all of the *N* samples were tested one-by-one. In each round, one sample was used for testing of the prediction model trained with all the other *N*−1 samples. After *N* rounds, all samples were tested one time, and the predicted tissue types were compared with the actual tissue types.

Since the positive and negative sample sizes were imbalance and Mathew’s correlation coefficient (MCC) can consider both sensitivity and specificity (Huang et al., 2015), MCC was used in IFS optimization. MCC can be calculated as follows:

(5) $$\text{MCC}=\frac{\text{TP}\times \text{TN}-\text{FP}\times \text{FN}}{\sqrt{\left(\text{TP}+\text{FP}\right)\,\left(\text{TP}+\text{FN}\right)\,\left(\text{TN}+\text{FP}\right)\,\left(\text{TN}+\text{FN}\right)}}$$

where TP, TN, FP, and FN stand for true positive, true negative, false positive, and false negative, respectively.

Based on the LOOCV MCC of each candidate gene set, an IFS curve can be plotted. The *x*-axis denoted the number of top genes that were used in the SVM classifier, and the *y*-axis denoted the LOOCV MCCs of the SVM classifiers. Based on the IFS curve, we can choose the right number of genes which had a good prediction performance as final biomarkers.

## Results and Discussion

### The Discriminative Importance of Genes

We applied mRMR algorithm to evaluate the discriminative importance of features iteratively. We want to find the features that were strongly associated with samples groups and were not redundant with other selected features. Using the mRMR method, we identified the top 100 most important genes. These genes were listed in Supplementary Table S1.

### The Optimal GBM Biomarker Genes Selected With IFS Method

**FIGURE 1:**
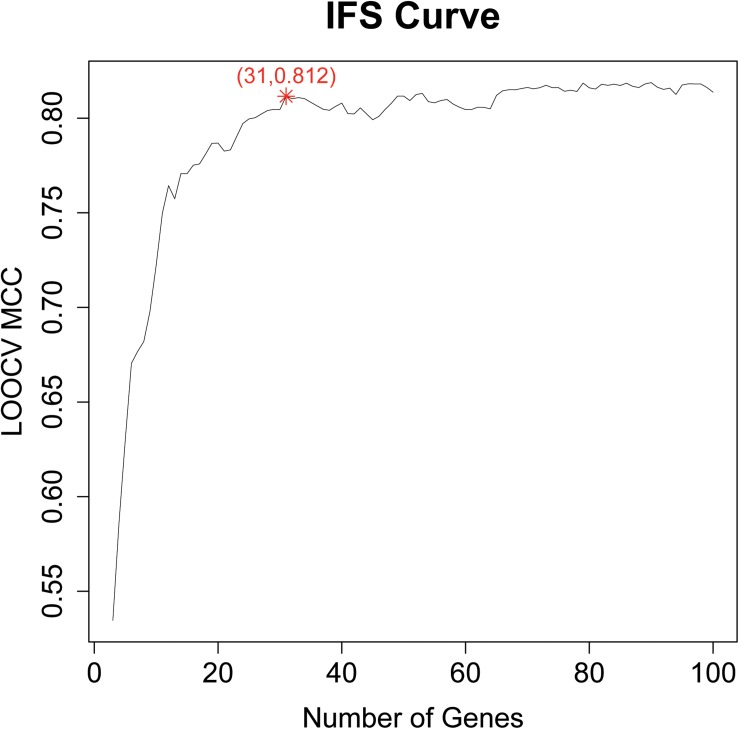
The IFS curve of the top 100 mRMR genes. The *x*-axis was the number of genes and the *y*-axis was the prediction performance, i.e., LOOCV MCC. The peak MCC was 0.812 when 31 genes were used. These 31 genes were selected as optimal GBM biomarker genes.

After we got the top 100 mRMR genes, we still did not know how many genes should be selected. To optimize the selected biomarker genes, we adopted IFS method. Each time, we added one feature into the previous feature set and got a new feature set. Then SVM classifiers were built to predict each sample’s labels during LOOCV. The IFS curve with the number of genes as *x*-axis and the prediction performance (LOOCV MCC) as *y*-axis were plotted in Figure 1. The peak MCC was 0.812 when 31 genes were used. These 31 genes were selected as optimal GBM biomarker genes. The 31 genes were listed in Table 1. The confusion matrix of the 31 genes were given in Table 2. The sensitivity, specificity, and accuracy were 0.948, 0.855, and 0.915, respectively.

**TABLE 1:** The 31 selected GBM biomarker genes.

Rank	Gene	Rank	Gene
1	TMSB4X	17	VIM
2	IPCEF1	18	ATP1A2
3	MTSS1	19	RPL41
4	S100A10	20	EGR3
5	HTRA1	21	OMG
6	DHRS9	22	LDHA
7	TPI1	23	P2RY12
8	SNX22	24	SPOCK1
9	FCGBP	25	NAMPT
10	TMSB10	26	C1QL2
11	CCL3	27	PTN
12	SLC6A1	28	CCL4
13	SMOC1	29	PDZD2
14	SEC61G	30	LGALS1
15	TGFBI	31	CLDN10
16	CDR1		

**TABLE 2:** The confusion matrix of the 31 selected genes.

	Predicted GBM	Predicted non-GBM
Actual GBM	2220	123
Actual non-GBM	181	1065

**FIGURE 2:**
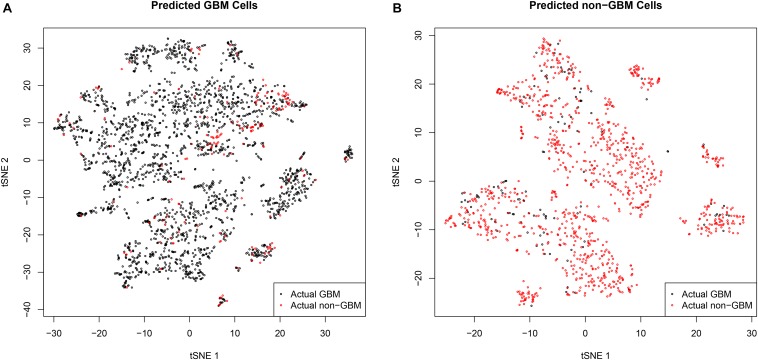
The *t*-SNE plots of predicted GBM cells and predicted non-GBM cells. **(A)** The *t*-SNE plots of predicted GBM cells. It can be seen that the false positive samples (red dots) and the true positive samples (black dots) were mixed and they were difficult to classify. **(B)** The *t*-SNE plots of predicted non-GBM cells. It can be seen that the false negative samples (black dots) and the true negative samples (red dots) were mixed. These *t*-SNE plots suggested that the GBM tissues may contain non-GBM cells and the non-GBM tissues may contain GBM cells, but most cells from the corresponding tissue were similar and the machine learning algorithm we used can get the robust single cell biomarkers even when there were tissue purity issues.

Since the tumor tissues are usually a mixture of tumor cells and normal cells, the tumor purity may cause the misclassifications. To check this, Figures 2A,B showed the *t*-distributed stochastic neighbor embedding (*t*-SNE) plots of predicted GBM cells and predicted non-GBM cells, respectively. In Figure 2A, it can be seen that the false positive samples (red dots) and the true positive samples (black dots) were mixed and they were difficult to classify. Similarly, in Figure 2B, it can be seen that the false negative samples (black dots) and the true negative samples (red dots) were mixed. These *t*-SNE plots suggested that the GBM tissues may contain non-GBM cells and the non-GBM tissues may contain GBM cells, but most cells from the corresponding tissue were similar and the machine learning algorithm we used can get the robust single cell biomarkers even when there were tissue purity issues.

### The Biological Functions of the Selected Genes

Upon analysis by the mRMR method, 31 important genes were extracted that may be essential biomarkers of GBM. We did Gene Ontology (GO) enrichment analysis of these 31 genes. The GO enrichment results were given in Table 3. It can be seen that their main function was cell adhesion and their main subcellular location was extracellular.

**TABLE 3:** The GO enrichment results of the 31 selected genes.

GO term	FDR	*P*-value	Genes
GO:0007155 cell adhesion	0.0068	8.26*E*−07	EGR3, LGALS1, OMG, PTN, S100A10, CCL4, SPOCK1, TGFBI, CLDN10, MTSS1, PDZD2, P2RY12
GO:0022610 biological adhesion	0.0068	8.74*E*−07	EGR3, LGALS1, OMG, PTN, S100A10, CCL4, SPOCK1, TGFBI, CLDN10, MTSS1, PDZD2, P2RY12
GO:0031012 extracellular matrix	0.0029	1.57*E*−06	LGALS1, OMG, HTRA1, PTN, SPOCK1, TGFBI, VIM, SMOC1
GO:0005615 extracellular space	0.0107	1.56*E*−05	LGALS1, OMG, HTRA1, PTN, CCL3, CCL4, SPOCK1, TGFBI, TMSB4X, TPI1, NAMPT
GO:0005576 extracellular region	0.0107	1.87*E*−05	ATP1A2, LDHA, LGALS1, OMG, HTRA1, PTN, S100A10, CCL3, CCL4, SPOCK1, TGFBI, TMSB4X, TPI1, VIM, FCGBP, NAMPT, PDZD2, SMOC1, C1QL2
GO:0005578 proteinaceous extracellular matrix	0.0107	2.30*E*−05	LGALS1, OMG, PTN, SPOCK1, TGFBI, SMOC1
GO:0044421 extracellular region part	0.0108	2.89*E*−05	ATP1A2, LDHA, LGALS1, OMG, HTRA1, PTN, S100A10, CCL3, CCL4, SPOCK1, TGFBI, TMSB4X, TPI1, VIM, FCGBP, NAMPT, SMOC1

We compared the 31 genes with reported GBM signatures in GeneSigDB (Culhane et al., 2012) and found that the 31 genes were significantly overlapped with a signature called “Human Glioblastoma_Morandi08_22genes” which were from Table 1 of Morandi et al. (2008): the 22 up-regulated genes following camptothecin (CPT) treatment in both U87-MG and DBTRG-05 cells. The hypergeometric test *p*-value was 0.0157.

Among the 31 genes, several of them plays roles in tumor metastasis. Thymosin β4 (TMSB4X/Tβ4) is associated with tumor metastasis and progression which plays a role in cell proliferation, migration, and differentiation through a TGFβ/MRTF Signaling Axis (Morita and Hayashi, 2018). TMSB4X expression was associated with cancers in a stage- and histology-specific manner and could be an effective prognostic parameter and prognostic index. Thus far, the relationship between TMSB4X and GBM remain unknown. IPCEF1 is the C-terminal half of CNK3 which is required for HGF-dependent Arf6 activation and migration during cancer metastasis (Attar et al., 2012). MTSS1 plays an important role in cancer metastasis. Previous researches indicated that MTSS1 as a potential tumor biomarker and its reduced expression associated with bad prognosis in many cancers. In GBM, MTSS1was reported as a potential tumor suppressor and prognostic biomarker which could suppress cell migration and invasion (Zhang and Qi, 2015).

Several genes can facilitate cancer progression. S100A10 is a calcium binding protein which is found to be significantly correlated with poor survival in patients with gliomas (Sethi et al., 2012). S100A10 has been involved in cancer progression, but the unique function is not well understood (O’Connell et al., 2010). HTRA1 encodes a ubiquitously expressed serine protease with prominent expression in the vasculature. Inhibition of HTRA1 could deregulate angiogenesis in the tumor stroma which plays an important role in tumor progression (Chien et al., 2006; He et al., 2010; Klose et al., 2018).

There are several other reported tumor genes. DHRS9 is a member of the short-chain dehydrogenases/reductases (SDR) family. Recent research found that SDR family members have been involved in tumors (Hu et al., 2016). TPI1 encodes an enzyme, consisting of two identical proteins, which catalyzes the isomerization of glyceraldehydes-3-phosphate (G3P) and dihydroxy-acetone phosphate (DHAP) in glycolysis and gluconeogenesis. TPI1 was down-regulated in response to LLL12 treatment and validated using immunoblot (Jain et al., 2015). It may serve as potential therapeutic targets in GBM (Jain et al., 2015).

## Conclusion

Glioblastoma is the most aggressive and incurable primary brain cancer in adults. The most common survival time after diagnosis is 12–15 months, with 5-year survival rate <5%. Symptoms of GBM are non-specific at early stage and the cause of GBM remains elusive. We analysis the data from 2343 tumor cells and 1246 periphery cells using mRMR and IFS method to characterize infiltrating tumor cells, and to define the cellular diversity.

## Data Availability Statement

The datasets generated for this study can be found in the https://www.ncbi.nlm.nih.gov/geo/query/acc.cgi?acc=GSE84465.

## Author Contributions

S-SF and QC conceived and designed the study. QC, JL, Z-YD, and S-SF performed the data mining and statistical analyses. FF, HC, and Z-YW prepared the figures and tables. QC and JL drafted the initial manuscript. S-SF made critical comments and revision for the initial manuscript. S-SF, QC, and JL had primary responsibility for the final content. All authors reviewed and approved the final manuscript.

## Conflict of Interest

The authors declare that the research was conducted in the absence of any commercial or financial relationships that could be construed as a potential conflict of interest.

## Supplementary Material

TABLE S1 The top 100 mRMR genes.
